# The Global One Health Paradigm: Challenges and Opportunities for Tackling Infectious Diseases at the Human, Animal, and Environment Interface in Low-Resource Settings

**DOI:** 10.1371/journal.pntd.0003257

**Published:** 2014-11-13

**Authors:** Wondwossen A. Gebreyes, Jean Dupouy-Camet, Melanie J. Newport, Celso J. B. Oliveira, Larry S. Schlesinger, Yehia M. Saif, Samuel Kariuki, Linda J. Saif, William Saville, Thomas Wittum, Armando Hoet, Sylvain Quessy, Rudovick Kazwala, Berhe Tekola, Thomas Shryock, Michael Bisesi, Prapas Patchanee, Sumalee Boonmar, Lonnie J. King

**Affiliations:** 1 Global Health Programs, College of Veterinary Medicine, The Ohio State University and VPH-Biotec Global Consortium, Columbus, Ohio, United States of America; 2 Department of Parasitology, Hôspital Cochin, Paris Descartes University, Paris, France; 3 Centre for Global Health Research, Brighton and Sussex Medical School, Sussex, United Kingdom; 4 College of Agricultural Sciences, Federal University of Paraiba, Brazil (CCA/UFPB), Areia, Paraiba, Brazil; 5 Department of Microbial Infection and Immunity, Center for Microbial Interface Biology, The Ohio State University, Columbus, Ohio, United States of America; 6 Food Animal Health Research Program, The Ohio State University, Wooster, Ohio, United States of America; 7 Centre for Microbiology Research, Kenya Medical Research Institute (KEMRI), Nairobi, Kenya; 8 Department of Pathology and Microbiology University of Montreal, Saint-Hyacinthe, Québec, Canada; 9 Faculty of Veterinary Medicine, Sokoine University of Agriculture, Chuo Kikuu, Morogoro, Tanzania; 10 United Nations Food and Agriculture Organization (FAO), Rome, Italy; 11 Elanco Animal Health, Greenfield, Indiana, United States of America; 12 The Ohio State University College of Public Health, Columbus, Ohio, United States of America; 13 Chiang Mai University, Chiang Mai, Thailand; 14 Thailand MOPH-U.S. CDC Collaboration, Bangkok, Thailand; George Washington University, United States of America

## Abstract

Zoonotic infectious diseases have been an important concern to humankind for more than 10,000 years. Today, approximately 75% of newly emerging infectious diseases (EIDs) are zoonoses that result from various anthropogenic, genetic, ecologic, socioeconomic, and climatic factors. These interrelated driving forces make it difficult to predict and to prevent zoonotic EIDs. Although significant improvements in environmental and medical surveillance, clinical diagnostic methods, and medical practices have been achieved in the recent years, zoonotic EIDs remain a major global concern, and such threats are expanding, especially in less developed regions. The current Ebola epidemic in West Africa is an extreme stark reminder of the role animal reservoirs play in public health and reinforces the urgent need for globally operationalizing a One Health approach. The complex nature of zoonotic diseases and the limited resources in developing countries are a reminder that the need for implementation of Global One Health in low-resource settings is crucial. The Veterinary Public Health and Biotechnology (VPH-Biotec) Global Consortium launched the International Congress on Pathogens at the Human-Animal Interface (ICOPHAI) in order to address important challenges and needs for capacity building. The inaugural ICOPHAI (Addis Ababa, Ethiopia, 2011) and the second congress (Porto de Galinhas, Brazil, 2013) were unique opportunities to share and discuss issues related to zoonotic infectious diseases worldwide. In addition to strong scientific reports in eight thematic areas that necessitate One Health implementation, the congress identified four key capacity-building needs: (1) development of adequate science-based risk management policies, (2) skilled-personnel capacity building, (3) accredited veterinary and public health diagnostic laboratories with a shared database, and (4) improved use of existing natural resources and implementation. The aim of this review is to highlight advances in key zoonotic disease areas and the One Health capacity needs.

## Introduction

Zoonotic infectious diseases have been important concerns to humans since the beginning of the domestication of animals 10,000 years ago. Infectious diseases remain a significant cause of mortality and morbidity globally; approximately 75% of emerging infectious diseases (EIDs) are zoonoses [1]. The phenomenon of emerging and reemerging infectious diseases is driven by various anthropogenic factors, including the following: genetic and biological factors, such as microbial adaptation to macro- and microenvironmental changes along with changes in host susceptibility to infection; environmental factors, including climate change, changes in ecosystems, and changes in human and animal population densities; and socioeconomic and political factors, such as increasing international travel and commerce, social inequality, poverty, conflict, famine, lack of political will, and changes in economic development and land use. In an Institute of Medicine report [2], the dynamics of these factors were referred to as a “convergence model,” with these forces creating the perfect microbial storm to accelerate the emergence of new infectious diseases.

Over the last 15 years, our planet has faced more than 15 deadly zoonotic or vector-borne global outbreaks, both viral (e.g., Hanta, Ebola, highly pathogenic avian influenza [H5N1 and recently H7N9], West Nile, Rift Valley fever, norovirus, severe acute respiratory syndrome [SARS], Marburg, influenza A [H1N1]) and bacterial (e.g., *Escherichia coli* O157:H7, *Yersinia pestis*, and *Bacillus anthracis*, the causes of hemolytic uremic syndrome, plague, and anthrax, respectively). Since 1980, more than 87 new zoonotic and/or vector-borne EIDs have been discovered.

The global economic burden due to zoonotic diseases is very high. According to a recent World Bank estimate, the economic burden due to six of the zoonotic diseases that have occurred in specific countries between 1997 and 2009 is estimated to be US$80,000,000,000 [3]. In a worst-case scenario, potential losses from a pandemic influenza outbreak could be US$3 trillion, which is equivalent to 5% of the global GDP. A recent report from the International Livestock Research Institute [4] highlighted zoonoses as major obstacles to poverty alleviation, affecting 1,000,000,000 livestock keepers. The report estimated that there are over 2,500,000,000 cases of human illness and 2.7 million deaths annually due to the top 56 zoonoses. In addition, the report estimated this group of diseases infected more than one in seven of the livestock found in less developed countries.

While zoonotic EIDs are a major concern globally, their impact in less developed countries is disproportionately high because of the occurrence of risk factors such as a high rate of population growth, lack of infrastructure and skilled-manpower capacity to tackle disease outbreaks, a high proportion of people with compromised immunity due to comorbidities such as HIV/AIDS or parasitic diseases, and lifestyles in which daily life depends on animals.

According to the United Nations Food and Agriculture Organization (FAO), global demand for meat production will increase from 233 million metric tons in the year 2000 to 300 million in 2020, with the major need being in less developed countries [5]. This trend will result in an increase in farms and the production of crops, with an expected increase in zoonotic food and vector-borne pathogens and environmental hazards. In response to the global need for prevention of diseases at the human, animal, and ecosystem interface, various academic, intergovernmental, and research centers are playing a central role. At the FAO, One Health has been integrated at the interdepartmental working group (IDWG) level to focus strategically on issues common to the domains of human health, animal health, and the environment. Key issues the FAO is focusing on include surveillance and disease intelligence, the need to improve biosecurity in production and marketing, and mechanisms to address socioeconomic incentives.

In September 2011, 360 scientists from 35 nations met at the UN Conference Center in Addis Ababa (Ethiopia) to inaugurate the First International Congress on Pathogens at the Human Animal Interface (ICOPHAI). Recently, the second ICOPHAI held in Porto de Galinhas, Brazil, (14–17 August 2013) attracted representatives of academic institutions and organizations from 59 different countries that presented 278 high-quality scientific studies. Key priority thematic areas that address One Health were presented, including the following: the interconnectedness of infectious diseases and One Health, the emergence and reemergence of vector-borne parasitic diseases and the role of the environment, respiratory diseases of global significance, food and waterborne diseases, the role of wildlife in newly emerging pandemics, drug development and antimicrobial resistance, environmental health, immunology and vaccine development, and One Health capacity-building needs. This review article highlights scientific advances, with a main emphasis on studies conducted in less developed nations.

## Key Highlights of One Health Issues and Global Impact

The current epidemic of Ebola virus in West Africa and the 2009 influenza A (H1N1) pandemic serve as stark reminders of the unpredictable nature of pathogens and the importance of animals in the ecology and emergence of viral strains. The ongoing pandemic of a disease such as tuberculosis (TB) is worrisome given a growing rate of highly resistant bacterial strains worldwide and infections in immunocompromised hosts such as those with HIV infection. Today TB results in an estimated 1.4 million deaths each year, and the worldwide number of cases (>9 million) is higher than at any time in history, despite the promising progress in the development of improved diagnostics, therapies, and vaccines and progress in identifying biomarkers of exposures and/or disease and associated host susceptibility [6], [7]. Although not frequently documented, animal–human interfaces exist, with transmission of *Mycobacterium bovis* among badgers, deer, bison, lions, and dogs to cattle, with spillover to and spillback from wildlife reservoirs. TB-complex mycobacteria were isolated at different rates (18%–82%) in wild animals [8], [9]. *M. bovis* strains from infected wildlife shared a similar molecular pattern with sampled livestock, suggesting that transmission between livestock and wildlife has occurred as reported by Gabriel and others [10], [11]. Transboundary migration and migration across continents are additional risk factors that also remain major means of transmission.

Foodborne parasitic, viral, and bacterial pathogens are responsible for an increasing burden of disease worldwide. Importantly, data indicating trends in foodborne infectious gastrointestinal disease are limited to a few industrialized countries, and even fewer data are available from low-resource developing regions. In 2010, over 21,000,000,000 food animals were produced to feed the world population. The urban population in sub-Saharan Africa is expected to double to nearly 800 million people by 2030 [12], and the resulting increase in population density will only exacerbate problems of transmission of virulent pathogens. Africa accounts for 41% of all the 1.8 million estimated diarrhea deaths among children in the developing world [13]. Physical infrastructure development in sub-Saharan Africa has not kept pace with massive urban in-migration, resulting in increased population density and the expansion of urban slums, creating a variety of public health challenges and the potential for catastrophic epidemics [14].

The majority of the parasitic diseases worldwide are also zoonotic, transmitted either directly between animal and human hosts or indirectly via consumption of raw food containing flesh from domestic or feral animals, consumption of food and water contaminated by animal or human feces, or via arthropod vectors, which can be impacted by ecological or climatic changes. Strengthening the veterinary surveillance and inspection infrastructure at the slaughterhouse is a highly important measure to detect foodborne parasites such as hydatid cysts (cysts of *Echinococcus granulosus*) in the livers of cattle, sheep, and camels and to prevent further transmission to dogs and humans. Other parasitic microorganisms such as *Cryptosporidium* can be a source of debilitating, prolonged diarrhea in immunosuppressed patients. The prevalence of cryptosporidiosis among HIV/AIDS patients is often high; according to Etsehiwot [10], the prevalence was 43.9%. The success of preventive measures will require an efficient network of public health intervention measures including routine surveillance for disease in humans and animals.

Vector-borne parasitic diseases also remain a major concern that requires the One Health approach for effective mitigation, control, and prevention. However, some of the vector-borne diseases such as tick-borne zoonoses are overshadowed by other major arthropod-borne diseases. For example, leishmaniasis, dengue fever, and malaria are largely unknown to communities, and often physicians fail to detect them, as highlighted by Szabo [11]. An interesting example of a public-private partnership (PPP) for the control of zoonotic vector-borne disease, called “Stamp Out Sleeping Sickness (SOS),” has been implemented in Uganda, a country that harbors both the acute and chronic forms of the human African trypanosomiasis. The SOS initiative facilitates control of human disease through an innovative community-based livestock spraying, indicating that a more holistic One Health approach is effective to control this fatal human disease. This PPP could be a model for other regions and diseases in order to develop a sustainable prevention and control system.

Antimicrobial resistance remains one of the leading public health issues globally and an added risk of zoonotic bacterial pathogens. Indeed, antimicrobial resistance is an emerging confounding clinical challenge, with 60% to 80% of recent *Salmonella* Typhimurium clinical isolates in Kenya being multidrug resistant. Recent studies clearly show that a multidrug-resistant haplotype that is rapidly expanding in Southeast Asia and that has been associated with fluoroquinolone resistance is now common and spreading across the African region as well [11], [15]. Managing the human health risks associated with antimicrobial-resistant bacteria in the food chain requires national, international, interdisciplinary, and intersectoral cooperation. The prudent use of antimicrobial agents in all sectors including humans, veterinary care, and horticulture was stated as a key measure that needs to be taken towards prevention and control of antimicrobial resistance [16], [17]. This is acknowledged by various intergovernmental organizations, such as the FAO, the World Health Organization (WHO), the World Organization for Animal Health (OIE), and the European Commission (EC), as well as professional associations and national authorities. Such prudence will not only safeguard the efficacy of antimicrobial drugs in veterinary medicine but, importantly, will prevent the emergence and spread of undesirable resistance phenotypes in pathogens transmissible between animals and humans.

Viruses belonging to at least 11 families are associated with foodborne transmission [18], [19]. Of these, nine are RNA viruses that are highly variable genetically due in part to low fidelity of the RNA-dependent RNA polymerases. Most are associated with diarrheal disease and can affect animals and humans. Noroviruses are extremely stable in the environment. While norovirus is the leading cause of foodborne disease in the United States, far exceeding estimates for bacterial pathogens [20] responsible for 58% of all cases (nearly 5.5 million illnesses a year in the US alone), investigations in low-resource developing country settings is very limited. The epidemiology of noroviruses and other enteric viruses clearly indicates the role of the host, pathogen, and environment, and the implementation of the One Health approach in tandem with other zoonotic disease control strategies would be instrumental.

## Capacity-Building Needs in Low-Resource Developing Countries

One of the most important aspects related to the control of pathogens at the human, animal, and environment interface is the development of adequate science-based risk management policies that respect transboundary regulations. Efforts have been incorporated in the principles of the tripartite initiative among intergovernmental organizations, with the FAO, WHO, and OIE to jointly pursue the One Health approach, working in close collaboration with research institutions, academia, intergovernmental organizations, the private sector, nongovernmental organizations, civil societies, and other stakeholders. An adequate surveillance system, including a strong laboratory network, is a key component of any meaningful prevention and control of zoonotic diseases. In order to develop an effective One Health implementation plan for strengthening capacity at national, regional, or global levels, there needs to be reexamination of how existing systems are structured, resourced, and managed. Such analyses will enable the development and sustainability of synergies among the human health, animal health, and ecosystem sectors [21]. Many of the studies presented at the ICOPHAI session on capacity building and One Health metaleadership were related to risk management and surveillance systems with specific insights and recommendations for operationalizing One Health. Inherent to overall surveillance is improved practice applying environmental monitoring methods to better characterize sources and profiles of environmental contaminants and pathways (i.e., air, food, and water) and modes (i.e., inhalation and ingestion) of both human and animal exposures to pathogenic agents. Summaries of the presentations and the key priorities are as outlined in Table 1.

**Table 1:** Summary of capacity building issues and origins of the studies reported at ICOPHAI.

Topic (Total Number of Studies)	ICOPHAI 2011 (Number of Studies)	ICOPHAI 2013 (Number of Studies)	Areas Covered	Countries
Food HACCP* (14)	2	12	Risk analysis, production practices, poultry production, surveillance, investigation, livestock/fishery production, implementation, and food security	South Africa, Kenya, Germany, Japan, US, Mozambique, Canada, and Germany
Milk HACCP (7)	1	6	Risk analysis, production and handling practices, consumption, implementation, and genetics	Côte d'Ivoire, Tanzania, Japan, Ethiopia, US, and The Netherlands
Government Implementation (4)	3	1	VPH use, biosecurity assessment, and health facility renovation/upgrading	Bangladesh and Ethiopia
One Health (6)	3	3	Development, implementation, strategies, VPH curriculum, and zoological parks	Cameroon, Portugal, Scotland, and US
Disease Surveillance and Outbreak Investigation (12)	3	9	Wildlife, livestock, Anthrax outbreak	Bolivia, Ethiopia, Uganda, Rwanda, and US
Other (12)	4	8	Veterinary diagnostic laboratory, immunology and vaccine development, surveillance, malaria preseason treatment, water safety, and HIV	Canada, US, England, Ethiopia, Italy, Mexico, Sudan, Brazil, and Germany

*HACCP = hazard analysis critical control point.

A plenary presentation from the FAO by Tekola [11] indicated that the FAO is applying One Health at the global level, translating theory into practice through outreach and engagement at the community level, and tapping into the technological innovation in mobile technology for animal disease surveillance through empowerment of local people to report real-time events in their communities, thus giving ownership of the solutions to their common concerns regarding health and disease, livelihoods and welfare, and learning and teaching. Another demonstration of the absolute importance of capacity building in the development and application of risk management policies was given by Sylvain Quessy and Ann Letellier [10], who underscored how training various stakeholders with complimentary roles, including policy makers and inspectors, was critical for the development of hazard analysis critical control point (HACCP)-based good production practices (GPPs) in an emerging-economy country like Vietnam. The application of GPPs not only improves the quality of life and health of people but also can help to increase economic incentives to producers and processors that further improve that quality of life. Capacity building is necessary for the establishment of an enhanced laboratory network to improve the efficacy of these practices in both domestic and export market aspects.

Increasing the number and distribution of educated and skilled personnel has been shown to be a major limitation in most developing regions. Studies demonstrated the high demand for appropriately and adequately educated and trained professionals and emphasized the capacity for the US and other developed-nations' institutions to provide comprehensive support to the less developed nations' academic institutes in various disciplines relevant to One Health. Education and training in risk analysis and all aspects related to the application of sanitary and phytosanitary standards (SPS)–related risk assessment capacities are highly needed. Overall, the crucial need for risk-based policy development is crucial before any attempt to operationalize One Health.

The systematic application of quality control systems following the WHO, FAO, and OIE guidelines are critical in ensuring risk management decisions. The development of laboratory networks linked with central reference laboratories associated with active and passive environmental and medical surveillance are essential. Surveillance is dependent on the availability of high-quality analytical and clinical laboratory diagnostic facilities. A clear illustration of the needs and benefits of an integrated surveillance system to better understand the emergence and epidemiology of zoonotic diseases, such as rabies, has been demonstrated. Better reporting systems from medical care facilities and a comprehensive national database that includes reports from environmental monitoring and human and animal health diagnostic systems are key components of an integrated surveillance system. This is deemed essential for One Health implementation. Collaborative regional networks that promote standardized curriculum for graduate studies within the framework of the One Health approach are currently emerging. One example is the SAPUVETNET, a partnership between Latin America and the European Union.

Needs for capacity building for scientists, extension officers, physicians, veterinarians, and technicians and better extension services at the farm or community level were also pointed out as urgent topics to improve rural economic development and capacity building. On-time and real-time communication and awareness creation to reach target audiences at the grassroots level and upward activities are implemented by the FAO through its established systems, of which Emergency Prevention System-Animal Health (EMPRES-AH), the Global Early Warning System for transboundary animal diseases and major zoonoses (GLEWS), and the Crisis Management Centre- Animal Health (CMC-AH) are a few examples [22]. Such systems play crucial roles in operationalizing One Health in low-resource settings.

Recognizing the serious consequences of EIDs dictates the need for development of thoughtful strategies to prevent and control diseases. Strategies have to take into account the economic, cultural, technological, and logistical issues encountered in developing countries. The development of capable diagnostic facilities is paramount for dealing with infectious diseases. Though some progress has been made, there is a need for continued investment and political commitment to meet the enormous persisting challenge to public health worldwide in the 21st century. We need more science to advance our knowledge in the complex interplay between microbe and host in pathogenesis, particularly with regard to resistant strains. We need improved in vitro and in vivo (animal) models to study basic, translational, and applied sciences related to diseases, especially in regards to understanding the complex interactions between a pathogen and its host in the context of immunobiology. There is a critical need for shorter, easier-to-deliver, safe, and low-cost antimicrobial regimens for resistant bacterial pathogens. There are ongoing complex changes to zoonotic and epidemic dynamics of TB and other infectious diseases in general. These changes impact the worldwide economy, ecosystems, and animal and human health. There is a greater need for One Health collaborative efforts that include the coordinated involvement of human and animal medicine as well as agriculture, wildlife, and environmental experts and policy makers in this arena.

## Final Remarks

ICOPHAI 2011 and 2013 were very successful in terms of information exchange on the scientific and policy aspects that enable operationalizing One Health worldwide. Within the two-year period, the number of the scientific presentations grew from 130 to 278, and the quality and depth of scientific presentations improved. However, the ultimate success comes only when the recommendations set forth are embraced and implemented by scientists and policy makers at the grassroots level. It has been very clear that convening a world forum comprised of multiple disciplines including academicians, researchers, and policy makers enriched the discussion and helped design ways for the implementation of One Health. To attain a true One Health approach, we need to recognize the interconnectivity among the health of humans and domestic or wild animals and the biotic and abiotic environment closely linked by the pathogens that they share. The congress delegates recognized that operationalizing One Health will enable collaborators to more effectively address and reduce the burden of zoonotic infectious diseases worldwide. To that end, the congress identified four areas for capacity-building needs, mainly directed at low-resource settings: (1) the development of adequate science-based risk management policies that respect transboundary and international guidelines, (2) sustained capacity building of applicably and appropriately knowledgeable and skilled One Health personnel, (3) accredited environmental and clinical diagnostic laboratories with an integrated and shared database, and (4) ensuring improved use of existing natural resources and implementation plans based on cost-benefit analyses. The findings obtained in the two ICOPHAI congresses encourage us to further promote One Health, and the forum will resume with its next congress in Chiang Mai, Thailand, in August 2015.

**Box 1. Key Learning Points**

- In order to successfully implement Global One Health, the development of adequate science-based risk management policies is essential.
- The global community has the responsibility for strengthening skilled manpower and infrastructure capacity in low-resource countries in order to effectively prevent and control diseases at the interface of human, animal, and environmental health and make the world a safer place.
- The Global One Health paradigm requires working across discipline and administrative barriers; this is a crucial component for effectively tackling complex One Health issues.

**Box 2. Top Five Papers in the Field**

- Coker R, Rushton J, Mounier-Jack S, Karimuribo E, Lutumba P, et al. (2011) Towards a conceptual framework to support One Health research for policy on emerging zoonoses. Lancet Infect Dis 11: 326–331.
- Smolinski MS, Hamburg MA, Lederberg J (2003) Microbial Threats to Health: Emergence, Detection, and Response. Washington (D.C.): National Academy Press.
- World Bank (2012) People, Pathogens and Our Planet. Volume 2, The Economics of One Health. World Bank Report (#69145-GLB). Washington (D.C.): World Bank. Available: http://documents.worldbank.org/curated/en/2012/06/16360943/people-pathogens-planet-economics-one-health. Accessed 6 October 2014.
- Grace D, Mutua F, Ochungo P, Kruska R, Jones K, et al. (2012) Mapping of poverty and likely zoonoses hotspots. Nairobi: International Livestock Research Institute.
- Miller M, Olea-Popelka F (2013) One Health in the shrinking world: Experiences with tuberculosis at the human-livestock-wildlife interface. Comp Immunol Microbiol Infect Dis 36: 263–268.
